# Risk Factors for Chronic Disease Among Rural Vietnamese Adults and the Association of These Factors With Sociodemographic Variables: Findings From the WHO STEPS Survey in Rural Vietnam, 2005

**Published:** 2007-03-15

**Authors:** Hoang Van Minh, Peter Byass, Dao Lan Huong, Nguyen Thi Kim Chuc, Stig Wall

**Affiliations:** Faculty of Public Health, Hanoi Medical University, Hanoi, Vietnam; Umeå International School of Public Health, Umeå University, Umeå, Sweden; Health Strategy and Policy Institute, Ministry of Health; Faculty of Public Health, Hanoi Medical University, Hanoi, Vietnam; Umeå International School of Public Health, Umeå University, Umeå, Sweden

## Abstract

**Introduction:**

Chronic diseases have emerged as a major health threat to the world's population, particularly in developing countries. We examined the prevalence of selected risk factors for chronic disease and the association of these risk factors with sociodemographic variables in a representative sample of adults in rural Vietnam.

**Methods:**

In 2005, we selected a representative sample of 2000 adults aged 25 to 64 years using the World Health Organization's STEPwise approach to surveillance of chronic disease risk factors. We measured subjects' blood pressure, calculated their body mass index (BMI), and determined their self-reported smoking status. We then assessed the extent to which hypertension, being overweight (having a BMI ≥25.0), smoking, and various combinations of these risk factors were associated with subjects' education level, occupational category, and economic status.

**Results:**

Mean blood pressure levels were higher among men than among women and increased progressively with age. The prevalence of hypertension was 23.9% among men and 13.7% among women. Sixty-three percent of men were current smokers, and 58% were current daily smokers; less than 1% of women smoked. Mean body mass index was 19.6 among men and 19.9 among women, and only 3.5% of the population was overweight. Education level was inversely associated with the prevalence of hypertension among both men and women and with the prevalence of smoking among men. People without a stable occupation were more at risk of having hypertension than were farmers and more at risk of being overweight than were farmers or government employees. Hypertension was directly associated with socioeconomic status among men but inversely associated with socioeconomic status among women.

**Conclusion:**

Rural Vietnam is experiencing an increase in the prevalence of many risk factors for chronic diseases and is in urgent need of interventions to reduce the prevalence of these risk factors and to deal with the chronic diseases to which they contribute.

## Introduction

Chronic diseases, including heart disease, stroke, cancer, diabetes, and chronic respiratory diseases, have emerged as a major health threat throughout the world but particularly in developing countries (1-4). Of the 58 million deaths that occurred worldwide in 2005, 35 million were attributable to chronic diseases, and 80% of these 35 million deaths occurred in developing countries (5). The annual number of deaths from chronic diseases is projected to increase to 41 million in the next 10 years, and most of these deaths will continue to occur in low- and middle-income countries (4,5).

The emerging chronic disease epidemics in developing countries can be explained largely by social and economic changes that have led to increases in the prevalence of risk factors for these diseases (6-8). And increases in the prevalence of major risk factors such as high blood pressure, tobacco use, physical inactivity, obesity, and alcohol consumption have been associated with a large portion of new cases of chronic diseases (9).

Evidence also shows that a large proportion of chronic disease cases are preventable and that the most cost-effective approach to containing emerging epidemics of chronic diseases is to reduce the prevalence of their risk factors (4,8,10,11). Because people who have major risk factors for chronic diseases are at greatly increased risk of developing chronic diseases in the future (12), the more we know about today's chronic disease risk factors, the better we will be able to control or prevent future chronic disease epidemics.

Vietnam, a developing country with a population of more than 83 million, is undergoing a rapid epidemiologic transition characterized by an increase in the prevalence of chronic diseases. According to national statistics, from 1986 to 2002, the proportion of all hospital admissions attributable to chronic diseases increased from 39% to 68%, and the proportion of deaths attributable to chronic diseases increased from 42% to 69% (13). To address this increase in chronic diseases, the Vietnamese Government issued Decision No 77/2002/QD-TTg (Ratification of Programme of Prevention and Control of Certain Non-communicable Diseases for the Period 2002–2010) (14), in which conducting research and surveillance and sharing epidemiologic information about chronic diseases were cited as urgently needed actions.

Though Vietnam has conducted some cross-sectional surveys, its health information system relies mainly on hospital-based statistics; however, these statistics describe only part of the nation's health situation, and Vietnam's policy makers and health managers need more population-based health data in order to make informed public health decisions. In 2005, to help provide such data, we conducted a study of chronic disease risk factors in the Bavi district of Vietnam. Using the STEPwise approach of the World Health Organization (WHO) (12), we examined the prevalence of three major preventable risk factors for chronic disease (high blood pressure, smoking, and being overweight) and the distribution of these risk factors by sociodemographic variables in a representative sample of adults in rural Vietnam.

## Methods

### Study setting and sample size

The Bavi district is a rural district located in northern Vietnam, about 60 km west of the capital, Hanoi. The district has a population of about 238,000; covers 410 square kilometers; and includes lowland, highland, and mountainous areas. Agricultural production and livestock breeding are the main economic activities of Bavi residents, whose average annual income is about US $78. The study described here was conducted in 2005 within the framework of a demographic surveillance system called FilaBavi (Epidemiological Field Laboratory of Bavi). A more detailed description of the Bavi district and of FilaBavi can be found elsewhere (15). The study was conducted in accordance with WHO’s STEPwise approach to surveillance of chronic disease risk factors (STEPS). STEPS involves three primary “steps”: 1) the use of a structured questionnaire to assess study subjects’ self-reported behavioral and lifestyle risk factors for chronic diseases, 2) the measurement of subjects’ blood pressure and anthropometrical parameters, and 3) the collection and biochemical analysis of subjects’ blood samples. Because STEPS is a standardized instrument that can be applied in various settings, STEPS data can be used to compare the health status of people in different regions of a country as well as that of people in different countries (12).

In this study, we implemented step 1 and step 2 in a representative sample of 2000 adults aged 25 to 64 years (approximately 250 in each of eight groups defined by sex and 10-year age range). Twelve field workers collected the data for the study after being trained in basic interviewing techniques and standard methods of obtaining physical measurements.

### Measurements

The data on smoking habits were based on participants' responses to questions in the tobacco use module of the STEPS questionnaire. The questions were designed to identify both current daily smokers and current nondaily smokers. Current daily smokers were defined as those who reported smoking at least one cigarette per day, and current nondaily smokers were defined as those who reported smoking less frequently.

Participants' blood pressure was measured three times with a standard digital sphygmomanometer (Omron Healthcare, Inc, Bannockburn, Ill) while they were in a sitting position after having rested for at least 5 minutes, and we used the average of the last two readings in our analyses. We considered subjects to have hypertension if their systolic blood pressure (SBP) was at least 140 mm Hg, their diastolic blood pressure (DBP) was at least 90 mm Hg, or they were being treated for hypertension (16).

Participants' weight and height were measured while they were without shoes and wearing light clothes. Their weight was measured to the nearest 10 g with electronic scales (Seca, Hamburg, Germany), and their height was measured to the nearest 0.1 cm with portable stadiometers. We then used these height and weight measurements to calculate participants' body mass index (BMI – their weight in kilograms divided by their height in meters squared) and considered anyone with a BMI of 25 or higher to be overweight (17).

We categorized study subjects by education level and occupation on the basis of their survey responses, and we categorized them by economic status on the basis of a previous evaluation by local authorities based in part on household rice production. Each of these sociodemographic variables had three categories. The education level categories were less than secondary school (completed less than 7 years of school), secondary school (completed 7 to 9 years of school), and high school or higher (completed more than 9 years of school); the three occupation categories were farmer, government employee, and other; and the three economic status categories were low, middle, and high.

### Data analysis

We produced both descriptive and analytical statistics using Stata 8 software (Stata Corp LP, College Station, Tex) and calculated means and proportions for variables of interest. We then used multivariate logistic regression to model the associations between our outcome variables (hypertension, smoking, overweight, and different combinations of these risk factors) and the sociodemographic factors previously described. We used 95% confidence intervals to determine whether associations were significant.

## Results

Of the 2000 subjects randomly selected from the FilaBavi study base, 1984 (987 men and 997 women) responded to the survey (response rate, 99.2%). The characteristics of the final study sample are described in Table 1.

Mean blood pressure levels were significantly higher among men than women. The mean SBP was 126.6 (95% CI, 125.4–127.9) among men vs 117.8 (95%, 116.7–118.9) among women, and the mean DBP was 77.0 (95% CI, 76.0–78.0) among men vs 72.5 (95% CI, 71.2–73.8) among women. Among men, mean blood pressure levels increased from 122.2 SBP (95% CI, 120.7–123.7) and 72.8 DBP (95% CI, 71.7–73.9) among those aged 25–34 to 132.2 DBP (95% CI, 129.3–135.2) and 80.4 SBP (95% CI, 78.6–82.3) among those aged 55–64; among women, mean levels increased from 111.4 DBP (95% CI, 110.1–112.7) and 67.7 SBP (95% CI, 66.6–68.8) among those aged 25–34 to 127.1 DBP (95% CI, 124.2–130.0) and 72.5 SBP (95% CI, 71.2–73.8) among those aged 55–64 (data not shown).

Table 2 (a and b) shows the distribution of selected major risk factors for chronic disease by sex and 10-year age group. The overall prevalence of hypertension in Bavi was 18.8% (23.9% among men and 13.7% among women). Of Bavi residents with hypertension, only 35.1% (37.8% of hypertensive men and 32.2% of hypertensive women) were aware of their hypertension, and only 20.1% (17.8% of the men and 24.1% of the women) were being treated for it. Smoking was the main form of tobacco use in Bavi and was very common among men. About 63% of men reported that they currently smoked, and 58% reported doing so daily. The prevalence of smoking among women was only 0.6%. Excess weight was not a major problem in Bavi. The mean BMI was 19.6 (95% CI, 19.3–19.9) among men and 19.9 (95% CI, 19.5–20.3) among women (data not shown), and the prevalence of overweight was only 3.0% among men and 4.0% among women. The most prevalent combination of risk factors was hypertension and smoking (7.2%), followed by hypertension and overweight (1.3%), overweight and current smoking (0.7%), and all three risk factors (0.4%).

We used multivariate logistic regression models to further analyze the association between selected risk factors for chronic diseases and the sociodemographic variables of age, education level, occupational category, and economic status. The risk factors analyzed were hypertension and overweight (among both men and women) and smoking and the combination of hypertension and smoking (among men only). As shown in Table 3, age was significantly associated with hypertension among both men and women and with the combination of hypertension and smoking among men. The prevalence of hypertension increased significantly with age, especially among women (ORs vs women aged 25–34 were 2.7, 5.3, and 11.7, respectively, among women in the next three age categories). Among men, age was also significantly associated with the prevalence of hypertension and smoking combined (ORs vs men aged 25–34 were 2.2, 2.0, and 3.7, respectively, among men in the next three age categories). However, we found no significant association between age and current smoking prevalence among men or between age and overweight among men or women.

In our multivariate analysis, we also found that among all men, those in the lowest education category were more likely to have hypertension than those in the highest (OR, 2.5) and that among men who smoked this association was only slightly weaker (OR, 2.1). Among women, occupation was related to hypertension and overweight: those in the "other" occupational category were significantly more likely to be hypertensive (OR, 1.7) and to be overweight (OR, 2.6) than were those who were farmers.

Interestingly, the relationship between economic status and hypertension among men differed substantially from that among women: whereas men in the low economic status group had a significantly lower risk for hypertension than those in the high group (OR, 0.4), women in the low and middle groups both had a significantly higher risk than those in the high group (ORs, 2.6 and 1.6, respectively). Men in the low and middle groups were more likely to currently smoke than were those in the high group (ORs, 2.0 and 1.4, respectively).

## Discussion

### Burden of major preventable chronic disease risk factors

The overall 18.8% prevalence of hypertension found in this study indicates that the condition already affects a large proportion of the adult population in the Bavi district and that the prevalence has increased substantially since 2002 when a STEPS survey of the same population indicated that the prevalence was only 14.1% (18). The prevalence was also higher than the prevalence of 16.8% found in a study by the Vietnam National Heart Institute in 2001 for both urban and rural areas in some provinces in the north of Vietnam (19) and the 16.9% nationwide prevalence among people aged 25–64 reported in the 2002 Vietnam National Health Survey (20). Internationally, similar findings about high and increasing rates of hypertension have also been reported in studies of rural communities in India (4,21), China (4), and Indonesia (22).

The results of our study show that Bavi residents with hypertension were more likely to be aware of their condition in 2005 than they were in 2002 (35% vs 17%) and that they were more likely to be receiving treatment for it (20% vs 7%) (18). These higher awareness and treatment rates could be due in part to the influences of the 2002 STEPS survey, during which Bavi residents with hypertension were told about their blood pressure status and given advice or referred to the district health center for a further health check. However, the higher awareness and treatment rates did not seem to have a marked impact on the hypertension problem in Bavi, indicating the need for a more comprehensive approach to dealing with hypertension.

The high prevalence of current smoking among men that we found in Bavi (63%) was slightly higher than the 56% found in previous studies in Bavi (23,24) or the 53% found in studies of smoking prevalence in Vietnam as a whole (20,25). Smoking prevalence has also been reported to be on the rise in other Asian countries, including China (4) and Indonesia (22). The findings from this study suggest that rural Vietnam is now at the latter stage of the smoking epidemic described by WHO (26) and that if the smoking epidemic model applies, rural Vietnam can be expected to experience a substantial increase in rates of smoking-related illness and death in the coming decades (27).

The findings from this study indicate that 14% of men in Bavi had hypertension and smoked. Because people with multiple risk factors are at significantly increased risk for cardiovascular disease (28,29) and because chronic diseases have been shown to be leading causes of death in Bavi (30-32), this high rate of multiple risk factors indicates an urgent need for comprehensive and integrated interventions to reduce the prevalence of cardiovascular disease and its risk factors in Bavi.

### Social patterning of risk factors for chronic diseases

Of particular interest are the associations we found between risk factors (hypertension, smoking, overweight, and combinations of these factors) and sociodemographic factors (sex, age, education level, occupation, and economic status).

Hypertension and smoking were each significantly more prevalent among men than among women. This finding is consistent with the results of previous studies in Vietnam (18-20,23-25). Despite the lower prevalence of hypertension and smoking rates among women, the danger that these risk factors pose for the cardiovascular health of women must not be underestimated, as hypertension and smoking have been shown to be strongly associated with coronary heart disease among women (33) as well as among men. The results of this study also confirm results from previous Vietnamese (18-20) and international studies (34) showing that age is a key predictor of hypertension.

We found that the prevalence of hypertension and the prevalence of multiple risk factors were both inversely associated with education among men, even after adjusting for other independent variables such as age and economic status. The inverse association between hypertension and education was also found in the previous local study (18) and in studies conducted in developed countries (35). In other developing countries, the pattern of the association between hypertension and education level varied; it was found to be inverse in China but direct in India (35). The rate of death from cardiovascular disease in Bavi from 1999 through 2003 was also significantly higher among less educated people (32).

In terms of occupation, women in government jobs were at significantly higher risk for hypertension than women who were farmers, possibly because of less physically active lifestyles, work pressure, and psychosocial stress. Further investigation of why these women had a relatively high prevalence of hypertension is needed.

Overall, we found hypertension to have a complex association with economic status. Among men, hypertension was highest among those categorized as being in the richest group, but among women, it was highest among those categorized as being in the poorest. The high rate of hypertension among the better-off men of Bavi may reflect their adoption of western lifestyles such as high-fat diets, less physical activity, higher alcohol consumption, and job stress. The relatively high prevalence of hypertension among poor women may reflect alternative risk factors in this setting, such as early undernutrition (35). In fact, in the past, Vietnamese people valued boys over girls and often took better care of boys. Research in Vietnam has shown that undernutrition rates were higher among girls than among boys (36).

### Limitations of the study

Because this was a cross-sectional study, the results cannot be considered as more than a snapshot, and they do not allow any assessment of trends. When comparing the prevalences of chronic disease risk factors from this study with those from other studies, one must consider that other factors might contribute to any observed differences, such as differences in the age of the study subjects, in how hypertension was defined, in when the studies were conducted, in the urban versus rural characteristics of the population, or in the instruments and procedures used to measure blood pressure.

For this article, we included only data on tobacco use, blood pressure, and physical activity because these measurements have been well validated in FilaBavi. We did not assess patterns in the prevalence of other important risk factors for chronic diseases, such as alcohol consumption and physical inactivity, because of the difficulty of standardizing results (e.g., converting quantities of alcohol consumed into standard drinks, capturing farming and nonfarming components of physical activity) and of analyzing the data (especially data on alcohol consumption and physical activity).

### Policy implications 

In summary, our findings suggest that rural Vietnam is undergoing a rapid epidemiological transition characterized by an increase in the prevalence of risk factors for chronic diseases and that different sociodemographic groups in the population have moved through the course of the transition to different extents. Our findings also show that actions to reduce levels of chronic disease risk factors in rural Vietnam are clearly urgent. The area needs comprehensive and integrated interventions designed to reduce these risk factors, including both primary and secondary approaches, as well as policy-level involvements. The highest priority should be put on primary prevention, as it has been shown to be the most cost-effective approach (4,8,10,11). The aim should be to make small reductions in the prevalence of smoking and hypertension in a large proportion of the population. The interventions should address all people in society, but should focus especially on disadvantaged groups.

This was a preliminary study of risk factors for chronic diseases in a rural setting in a transitional country. Further studies over longer periods of time and deeper analyses will be required to give greater insights into the epidemiology of chronic diseases in such settings.

## Figures and Tables

**Table 1:** Selected Sociodemographic Characteristics of Study Sample, Bavi District, Vietnam, 2005

**Characteristic**	**Men (N = 987) n (%)**	**Women (N = 997) n (%)**	**Total (N = 1984) n (%)**
**Age, y**			
25-34	241 (24.4)	263 (26.4)	504 (25.4)
35-44	261 (26.4)	241 (24.2)	502 (25.3)
45-54	238 (24.1)	254 (25.5)	492 (24.8)
55-64	247 (25.0)	239 (24.0)	486 (24.5)
**Education level**			
Less than secondary school (<7 years)	263 (26.6)	358 (35.9)	621 (31.3)
Secondary school (7-9 years)	536 (54.3)	482 (48.3)	1018 (51.3)
High school or more (≥9 years)	188 (19.0)	157 (15.7)	345 (17.4)
**Occupation**			
Farmer	591 (59.9)	763 (76.5)	1354 (68.2)
Government employee	31 (3.1)	35 (3.5)	66 (3.3)
Other	365 (37.0)	199 (20.0)	564 (28.4)
**Economic status^a^**			
Low	113 (11.5)	118 (11.9)	231 (11.7)
Middle	652 (66.3)	643 (64.8)	1295 (65.5)
High	219 (22.3)	231 (23.3)	450 (22.8)

a Subjects' economic status is based on a previous assessment by local authorities that reflected subjects' household rice production during the previous year as well as a qualitative assessment of their status. Because of incomplete data for some subjects, percentages for this characteristic are based on a sample of 984 men, 992 women, and 1976 total.

**Table 2a:** Estimated 2005 Prevalence of Selected Risk Factors for Chronic Disease Among Male Bavi District Residents, by Age Group

**Risk Factor**	**Aged 25-34 y, % (95% CI*)***	**Aged 35-44 y, % (95% CI)**	**Aged 45-54 y, % (95% CI)**	**Aged 55-64 y, % (95% CI)**	**All Men Aged 25-64 y, % (95% CI)**
Hypertension^a^	10.0 (6.2-13.8)	21.5 (16.4-26.5)	25.6 (20.0-31.2)	38.5 (32.4-44.6)	23.9 (21.2-26.6)
Aware of having hypertension^b^	21.4 (10.3-32.5)	21.3 (10.7-31.9)	37.2 (27.3-47.2)	29.4 (23.5-35.2)	37.8 (23.9-61.7)
Receiving treatment for hypertension^b^	16.7 (7.8-18.5)	7.1 (3.5-64.6)	11.5 (4.1-85.2)	28.4 (4.7-91.8)	17.8 (2.5-72.7)
Current smoking	62.9 (56.8-69.1)	72.3 (66.8-77.8)	61.3 (55.1-67.6)	54.7 (48.4-60.9)	62.9 (59.9-66.0)
Current daily smoking	56.4 (50.1-62.7)	67.4 (61.7-73.2)	55.0 (48.7-61.4)	52.2 (46.0-58.5)	58.0 (54.9-61.0)
Overweight^c^	2.1 (0.3-3.9)	2.7 (0.7-4.7)	3.8 (1.3-6.2)	3.6 (1.3-6.0)	3.0 (2.0-4.1)
Hypertension and current smoking	7.5 (4.1-10.8)	14.2 (9.9-18.4)	13.0 (8.7-17.3)	21.9 (16.7-27.1)	14.2 (12.0-16.4)
Hypertension and overweight	0.4 (0.0-1.2)	1.5 (0.0-3)	2.5 (0.5-4.5)	2.8 (0.8-4.9)	1.8 (1.0-2.7)
Overweight and current smoking	1.2 (0.0-2.7)	0.8 (0.0-1.8)	1.3 (0.0-2.7)	2.0 (0.3-3.8)	1.3 (0.6-2.0)
Hypertension, current smoking, and overweight	0.4 (0.0-1.2)	0.4 (0.0-1.1)	0.4 (0.0-1.2)	1.6 (0.0-3.2)	0.7 (0.2-1.2)

CI indicates confidence interval

a Defined as having a systolic blood pressure ≥140 mm Hg, a diastolic blood pressure ≥90 mm Hg, or a diagnosis of hypertension.

b Percentage estimates for subset of the population with hypertension.

c Defined as having a body mass index ≥25.0.

**Table 2b:** Estimated 2005 Prevalence of Selected Risk Factors for Chronic Disease Among Female Bavi District Residents, by Age Group, and Among All Residents Aged 25–64

**Risk Factor**	**Aged 25-34 y, % (95% CI)**	**Aged 35-44 y, % (95% CI)**	**Aged 45-54 y, % (95% CI)**	**Aged 55-64 y, % (95% CI)**	**All Women Aged 25-64 y, % (95% CI)**	**All Residents Aged 25-64 y, % (95% CI)**
Hypertension^a^	3.4 (1.2-5.6)	7.9 (4.5-11.3)	14.6 (10.2-18.9)	30.1 (24.3-36.0)	13.7 (11.6-15.9)	18.8 (17.1-20.5)
Aware of having hypertension^b^	31.6 (8.6-54.6)	29.7 (14.3-45.2)	37.1 (25.5-48.7)	37.0 (28.8-45.3)	32.2 (27.4-36.9)	35.1 (22.6-56.7)
Receiving treatment for hypertension^b^	44.4 (17.6-106.6)	15.8 (8.6-107.5)	18.9 (6.5-97.5)	26.4 (5.2-91.3)	24.1 (3.7-79.6)	20.1 (16.0-71.8)
Current smoking	0.8 (0-1.8)	0.4 (0-1.2)	0.8 (0-1.9)	0.4 (0-1.2)	0.6 (0.1-1.1)	31.6 (29.6-33.7)
Current daily smoking	0.8 (0-1.8)	0 (0-0)	0.8 (0-1.9)	0.4 (0-1.2)	0.5 (0.1-0.9)	29.1 (27.1-31.1)
Overweight^c^	2.3 (0.5-4.1)	3.7 (1.3-6.1)	5.9 (3.0-8.8)	4.2 (1.6-6.7)	4.0 (2.8-5.2)	3.5 (2.7-4.3)
Hypertension and current smoking	0 (0-0)	0.4 (0-1.2)	0 (0-0)	0.4 (0-1.2)	0.2 (0.1-0.5)	7.2 (6.0-8.3)
Hypertension and overweight	0 (0-0)	0 (0-0)	0.8 (0-1.9)	2.5 (0.5-4.5)	0.8 (0.2-1.4)	1.3 (0.8-1.8)
Overweight and current smoking	0 (0-0)	0 (0-0)	0 (0-0)	0 (0-0)	0 (0-0)	0.7 (0.3-1.0)
Hypertension, current smoking, and overweight	0 (0-0)	0 (0-0)	0 (0-0)	0 (0-0)	0 (0-0)	0.4 (0.1-0.6)

CI indicates confidence interval.

a Defined as having a systolic blood pressure ≥140 mm Hg, a diastolic blood pressure ≥90 mm Hg, or a diagnosis of hypertension.

b Percentage estimates for subset of the population with hypertension.

c Defined as having a body mass index ≥25.0.

**Table 3:** Results of a Multivariate Analysis of Selected Risk Factors for Chronic Disease Among Bavi District Residents in 2005, by Sex, Age, Education Level, Occupation, and Economic Status

**Risk Factor**	**Hypertension**		**Smoking**	**Overweight**		**Hypertension and Smoking**

	**Men OR (95% CI)**	**Women OR (95% CI)**	**Men OR (95% CI)**	**Men OR (95% CI)**	**Women OR (95% CI)**	**Men OR (95% CI)**
**Age, y**						
25-34	Ref	Ref	Ref	Ref	Ref	Ref
35-44	2.7 (1.6-4.5)	2.7 (1.2-6.1)	1.6 (1.1-2.4)	1.0 (0.3-3.5)	1.9 (0.6-6.0)	2.2 (1.2-4.1)
45-54	3.4 (1.2-4.8)	5.3 (2.5-11.2)	1.0 (0.7-1.5)	1.1 (0.3-3.7)	3.0 (1.1-8.7)	2.0 (1.1-3.7)
55-64	3.8 (1.6-5.2)	11.7 (5.5-24.8)	0.7 (0.5-1.1)	0.7 (0.2-2.5)	1.6 (0.5-5.3)	3.7 (2.1-6.5)
**Education level**						
Less than secondary school (<7 years)	2.5 (1.5-4.1)	0.9 (0.5-1.7)	0.9 (0.6-1.4)	1.4 (0.4-4.7)	0.7 (0.2-2.2)	2.1 (1.2-3.8)
Secondary school (7-9 years)	1.8 (1.1-2.8)	0.8 (0.4-1.4)	0.8 (0.6-1.2)	1.2 (0.4-3.6)	1.2 (0.4-3.1)	1.3 (0.7-2.3)
High school or more (≥9 years)	Ref	Ref	Ref	Ref	Ref	Ref
**Occupation**						
Farmer	Ref	Ref	Ref	Ref	Ref	Ref
Government staff	1.8 (0.7-4.8)	2.3 (0.7-7.0)	0.4 (0.2-0.8)	1.0 (0.1-9.9)	0.9 (0.1-8.3)	1.4 (0.4-4.5)
Other	1.2 (0.8-1.7)	1.7 (1.1-2.7)	1.2 (0.9-1.7)	2.0 (0.9-4.6)	2.6 (1.2-5.8)	1.2 (0.8-1.8)
**Economic status^a^**						
Low	0.4 (0.2-0.8)	2.6 (1.3-5.2)	2.0 (1.2-3.4)	0.4 (0.2-1.5)	0.3 (0.1-2.7)	0.8 (0.4-1.7)
Middle	0.8 (0.6-1.2)	1.6 (1.3-2.7)	1.4 (1.1-2.0)	0.6 (0.3-1.4)	0.6 (0.1-2.9)	0.4 (0.3-1.4)
High	Ref	Ref	Ref	Ref	Ref	Ref

OR indicates odds ratio; CI, confidence interval; and Ref, referent group.

a Subjects' economic status is based on a previous assessment by local authorities that reflected subjects' household rice production during the previous year as well as a qualitative assessment of their status.
